# The Surgical Treatment of Nasal Catarrh

**Published:** 1888-11

**Authors:** A. B. Thrasher

**Affiliations:** Cincinnati, Ohio


### THE SURGICAL TREATMENT OF NASAL CATARRH.

BY A. B. THRASHER, M. D., CINCINNATI, O.

There are forms of nasal catarrh that can only be benefited, and not cured, by medicinal treatment. When we have mechanical obstruction to the lumen of the naris of a hypertrophic, hyperplastic or neoplastic character, surgery offers the most direct path to the removal of the difficulty. These are the cases of nasal catarrh which have passed from one doctor to another, have used all the advertised remedies, so-called, and have at last concluded that nasal catarrh is incurable, and it is no use to treat it.

The symptoms to which these conditions give rise are very varied, and many of them seem to have no relation to the causal rhinopathy. The surgical removal of the pathological growth has alone determined the cause of many obscure symptoms.

Since the cavity of the nose is open to inspection, directly and indirectly, it is rather curious that surgeons have been so slow to invade this region. They will cut with impunity into the cavity of the abdomen and remove or examine the contained viscera, or open the thorax and tie an artery, or evacuate an abscess, or even cut into the brain-box to elevate a depressed bone or remove a tumor, and yet stand with folded hands and do nothing to check the symptoms caused by a neoplasm springing from the middle turbinated, or a hypertrophy at the pharyngeal vault. This is not so curious when we stop to think that the removal of a growth from the posterior middle turbinated body requires more manual dexterity and tactile skill than to open the abdomen, thorax, or even the cranium itself.

When we meet with a simple enlargement of the turbinated body, which it is desirable to remove or reduce, what is the best method of procedure? There are cases in which the application of a chemical caustic answers the purpose admirably well. As a rule, however, the judicious and skillful use of some surgical instrument will accomplish the result more neatly, more rapidly and more satisfactorily.

The Woakes gouge or plow will do the work, and by the aid of cocaine will do it rapidly and painlessly. However, there is, as a rule, very considerable hemorrhage, and there is always a large scar surface. This scar surface not being covered with normal epithelium will afford a lodgment for crusts, which are a source of annoyance by obstructing the respiration and by decomposing, give rise to a bad odor. There is also an appearance of roughness in the use of the gouge, and a nervous patient might be very easily frightened, so that he would not submit to the operation after having seen the instruments, or having had the operation explained.

The cold wire snare, like the Jarvis or the Allen, is a much neater instrument, and for some conditions meets all the requirements. The snare may be used with or without the transfixing needle, and will nicely remove the hypertrophic mass. However, the same broad scar will result as after the use of the gouge. Where there is a polypoid projection from the surface, the snare is, as a rule, the last instrument to apply. Even though the projecting tumor be cartilaginous or even osseous, yet a No. 8 piano wire will generally accomplish its removal. In the removal of fibroids care must be taken in drawing the wire so as not to excise them too rapidly for fear of hemorrhage.

In uncomplicated cases of chronic hypertrophic rhinitis, the conditions to be met are: (a) Obstruction to lumen of naris, and (b) abnormal condition of mucous glands. The causal indications would at once suggest a removal of the redundant tissue. The means employed should look to the accomplishment of this with the least resulting scar surface, so that the natural condition of the epithelial covering might be as nearly as possible simulated. To reach this result, I have met with the most marked success in the use of the galvano-cautery knife. I first anæsthetize the part with cocaine; then I introduce the knife to the posterior part of the hypertrophy, turn the sharp edge toward the tissue to be cut, heat the knife white hot, and cut deeply, drawing the knife forward. The result is a deep linear scar in an antero-posterior direction through the hypertrophic tissue. I cut deeply enough to destroy a portion of the sub-mucous erectile tissue. When this cut heals the result is a narrow linear scar, and by the natural contraction of the cicatricial tissue the surface scar becomes in time partially obliterated.

Should the hypertrophy be great the operation can be repeated, making the cuts parallel until the part has been sufficiently reduced. This method of operating requires a powerful battery and a steady hand, and possesses many advantages over the application of the flat surface of the knife heated to a cherry red. The flat blade destroys the epithelium over a large surface, very like glacial acetic or tri-chlor–acetic acid, while the sharp edge destroys but a narrow strip. It is difficult to control the heat to an exact cherry red, and an increased current may cause a deep destruction of tissue with a conseqent broad eschar. This danger is entirely obviated in making the linear incision. The deep cut renders hemorrhage more probable, but as a rule a drainage from the cavernous tissue in this region will prove advantageous. Yet even this may be avoided, if thought necessary, by the withdrawal of the knife from the tissue before it has cooled.

By carefully reducing the hypertrophic tissue in this, or a similar manner, most of the obstinate cases of chronic nasal catarrh will recover, and in time the troublesome symptoms will subside.

Of course, proper constitutional and hygienic treatment must also be instituted in accordance with the conditions of the individual case.

